# In-hospital DVT detection, thromboprophylaxis practice, and guideline adherence in patients with traumatic fractures: a single-center retrospective study

**DOI:** 10.1016/j.rpth.2026.106926

**Published:** 2026-08-27

**Authors:** Hongjian Zeng, Yangyubo Zhang, Xianfeng Chen, Yong Zeng

**Affiliations:** Emergency Department, Jiangnan Campus, The Second Affiliated Hospital of Chongqing Medical University, Chongqing, China

**Keywords:** bone, fractures, risk assessment, venous thrombosis

## Abstract

**Background:**

Deep vein thrombosis (DVT) is a major complication in patients with traumatic fractures. Although clinical guidelines recommend risk stratification and appropriate thromboprophylaxis, the burden of in-hospital detected DVT remains high.

**Objective:**

To evaluate in-hospital DVT detection, the discriminative ability of the modified Caprini score, and thromboprophylaxis guideline adherence in hospitalized patients with traumatic fractures.

**Methods:**

This retrospective real-world study included 13,990 patients with traumatic fractures admitted to the Second Affiliated Hospital of Chongqing Medical University from January 2019 to October 2024. Demographic characteristics, comorbidities, fracture characteristics, and clinical data were extracted from electronic medical records. Patients were stratified into very-low-risk, low-risk, moderate-risk, and high-risk groups according to the modified, retrospectively reconstructed Caprini Risk Assessment Model, and thromboprophylaxis practice across risk categories was analyzed.

**Results:**

Among 13,990 hospitalized patients with traumatic fractures, DVT was detected during hospitalization in 1765 patients (12.62%). In the operational guideline-adherence analysis, restricted to patients for whom routine thromboprophylaxis was recommended and excluding patients whose prophylaxis was initiated only after DVT detection, 13,156 patients were included; 8121 (61.73%) received recorded pharmacological or mechanical thromboprophylaxis, and 6065 (46.10%) received guideline-concordant prophylaxis. Thromboprophylaxis use increased with higher risk categories, whereas overall guideline adherence remained suboptimal. The modified Caprini score showed moderate discrimination for in-hospital detected DVT.

**Conclusion:**

Approximately one in 8 hospitalized patients with traumatic fractures had DVT detected during hospitalization. Significant gaps in thromboprophylaxis practice and guideline adherence persist in real-world clinical settings. Improved risk assessment and optimized implementation of guideline-based thromboprophylaxis may help improve real-world VTE prevention practice in patients with traumatic fractures.

## Essentials

• Hospitalized patients with traumatic fractures have a high risk of deep vein thrombosis.
• This study evaluated deep vein thrombosis detection, thromboprophylaxis, and guideline adherence.
• Guideline-recommended thromboprophylaxis was underused in eligible patients.
• Improving guideline adherence may help prevent thrombosis after traumatic fractures.

## 1 Introduction

Venous thromboembolism (VTE), encompassing deep vein thrombosis (DVT) and pulmonary embolism (PE), is a major contributor to the global disease burden [1]. Previous studies have reported a substantial increase in VTE-related hospitalizations over recent decades [2].

The reported incidence of DVT in patients with traumatic fractures ranges from 9.1% to 61.0%, which is substantially higher than that in general surgical populations [3]. Given the large number of patients with traumatic fractures worldwide, fracture-related DVT imposes a considerable clinical and economic burden [4,5]. Therefore, effective thromboprophylaxis is essential for reducing VTE-related morbidity and healthcare burden in this population.

Early thromboprophylaxis can effectively reduce the risk of VTE, highlighting the importance of accurate risk stratification and timely intervention. Current guidelines emphasize structured VTE risk assessment and appropriate thromboprophylaxis; however, their recommendations differ in scope. The Chinese guideline for perioperative VTE prevention in trauma and fracture patients incorporates Caprini-based risk assessment in this clinical context, whereas the ACCP 9th edition guideline provides thromboprophylaxis recommendations for major orthopedic surgery patients but does not specifically mandate Caprini scoring for all traumatic fracture patients [6,7]. Previous studies have shown that 3.1% to 17.2% of orthopedic patients still develop DVT despite Caprini-based risk assessment and preventive measures [8,9], suggesting that the discriminative performance and clinical applicability of the Caprini score in fracture populations may require further evaluation.

Despite well-established guidelines for VTE prevention, adherence to thromboprophylaxis recommendations in real-world practice remains suboptimal. A multicenter study reported that only 55.8% of high-risk hospitalized patients received appropriate pharmacological thromboprophylaxis, although this represented a substantial improvement compared with earlier reports [10,11]. Concerns regarding bleeding risk, underestimation of VTE risk, and uncertainty about the risk–benefit balance of prophylaxis may contribute to inadequate implementation of guideline-based thromboprophylaxis in clinical practice [12].

Therefore, this study aimed to determine the proportion of hospitalized patients with traumatic fractures who had DVT detected during hospitalization, evaluate the discriminative ability of the modified Caprini score in this population, and assess adherence to guideline-based pharmacological and mechanical thromboprophylaxis. These findings may help identify gaps in current thromboprophylaxis practice and inform strategies to improve guideline-based VTE prevention in clinical practice.

## 2 Methods

### 2.1 Ethical statement

This retrospective single-center observational study included patients with traumatic fractures who were admitted to the Second Affiliated Hospital of Chongqing Medical University from January 1, 2019, to October 31, 2024, and met the predefined inclusion and exclusion criteria. The study was approved by the institutional review board of the second affiliated hospital of Chongqing Medical University, which waived the requirement for informed consent because of the retrospective study design. All data were anonymized and used exclusively for research purposes. The study was conducted in accordance with the Declaration of Helsinki and China’s Good Clinical Practice guidelines.

### 2.2 Patient inclusion and exclusion criteria

The inclusion criteria were as follows: (1) age ≥18 years and (2) a confirmed diagnosis of traumatic fracture. Patients were excluded if they were younger than 18 years or if diagnostic information was insufficient to confirm the diagnosis of traumatic fracture. Missing height, weight, or other nonessential covariates did not by itself result in exclusion from the study. Traumatic fractures were identified based on diagnostic coding and documented injury information in the medical records. All consecutive eligible patients during the predefined study period were included.

DVT events were identified using diagnostic records and venous compression ultrasonography reports obtained during hospitalization. Diagnostic records, including diagnosis entries or diagnostic codes, were used to screen potential DVT events; however, DVT events were included only when supported by venous compression ultrasonography findings. The ultrasonographic diagnostic criteria included complete or partial incompressibility of the venous lumen, absence of or filling defects in venous blood flow signals, and characteristic echogenic intraluminal thrombi [13]. Universal lower-extremity venous ultrasonography screening was not routinely performed for all patients at admission. In routine clinical practice, some patients underwent lower-extremity venous ultrasonography within 24 hours after admission, whereas subsequent ultrasonography was generally performed when patients developed symptoms suggestive of DVT or when DVT was clinically suspected.

Accordingly, the primary outcome was lower-extremity DVT detected during hospitalization, confirmed by venous compression ultrasonography and including both proximal and distal DVT. Because ultrasonography was not performed systematically at admission and thrombus chronicity could not be reliably established from the retrospective records, all ultrasonography-confirmed DVTs were included and were not analyzed separately by chronicity. The timing of thrombus onset was therefore not inferred from the time of detection. Pulmonary embolism was analyzed separately and was not included in the DVT outcome. Because symptom status was not consistently documented, DVTs were not classified as symptomatic or asymptomatic.

### 2.3 Data collection

Clinical data were extracted from the electronic medical record system. Variables required for Caprini risk assessment included demographic characteristics, comorbidities, mobility status, surgical factors, thrombosis history, thrombophilia-related factors, medication history, and fracture characteristics. In addition, data on in-hospital anticoagulant use were collected.

Age, diagnostic information, medical orders, and laboratory data were generally complete among the included patients. Missing information most commonly involved height, weight, and certain past medical history variables because of incomplete narrative documentation. Histories of hypertension, diabetes mellitus, and coronary artery disease were supplemented using diagnostic records when available. A history of VTE was retained because of its strong clinical relevance and important weighting within the Caprini score. Because height and weight were missing or considered unreliable in more than 30% of patients, BMI was not included in the modified Caprini score. Missingness in these variables did not result in patient exclusion.

For all analyses, the time origin was defined as the date and time of hospital admission. DVT detection time was defined as the time of the first venous compression ultrasonography examination showing DVT. The initiation time of pharmacological thromboprophylaxis was defined as the first recorded medical order time for anticoagulant prophylaxis, and the initiation time of mechanical thromboprophylaxis was defined as the first recorded order time for mechanical prophylaxis, including graduated compression stockings or intermittent pneumatic compression. For the thrombus-location analysis, proximal DVT was defined as involvement of the iliac, femoral, or popliteal veins. Distal DVT was defined as thrombosis confined to calf veins below the popliteal vein, including anterior tibial, posterior tibial, peroneal/fibular, gastrocnemius/soleal, or intermuscular calf veins. When both proximal and distal venous segments were involved, the event was classified as proximal involvement.

Because this was a retrospective real-world study, thromboprophylaxis status reflected clinical practice during hospitalization and was not assumed to represent prophylaxis exposure before DVT detection in all patients. Therefore, comparisons of DVT detection according to prophylaxis status were interpreted descriptively rather than causally, as prophylaxis was not modeled as a time-dependent exposure and residual confounding by indication could not be eliminated.

To reduce information bias, variables identified from diagnostic codes were extracted according to predefined diagnostic and classification criteria. ICD-based identification was mainly used for traumatic fracture diagnosis, fracture location, comorbidities, and severe traumatic brain injury. Key variables were further checked against clinical documentation, imaging reports, operation records, or medical orders when applicable. DVT events were not defined by diagnostic codes alone and were included only when supported by venous compression ultrasonography findings. Severe traumatic brain injury was defined using International Classification of Diseases codes and relevant literature, including diffuse brain injury (NA07.3), traumatic intracranial hemorrhage (NA07.1, NA07.5–NA07.8), and other or unspecified intracranial injuries (NA07.Y, NA07.Z) [14,15].

Patients were categorized according to fracture location into thoracic and trunk fractures (including clavicular, rib, chest, and spinal fractures), upper extremity fractures (including shoulder, humeral, and forearm fractures), combined thoracic/trunk and upper extremity fractures, pelvic fractures, femoral fractures, lower leg fractures (including tibial, fibular, and tibiofibular fractures), patellar fractures, foot fractures, multiple fractures involving the lower extremities and pelvis, and fractures at other sites (including head, facial, and hand fractures). These categories were summarized in Table 1, with detailed classification criteria provided in Supplementary Table 1.

**Table 1:** Baseline characteristics of patients

Characteristic	Overall (*N* = 13,990)	Low-risk and very low-risk group (*n* = 2440)	Moderate-risk group (*n* = 3081)	High-risk group (*n* = 8469)	*P* value^a^
Age, y	61.0 ± 17.92	47.0 ± 14.38	63.0 ± 13.49	63.0 ± 18.55	< .001
Sex					< .001
Male, *n* (%)	7326 (52.37%)	1552 (63.61%)	1497 (48.59%)	4277 (50.50%)	
Female, *n* (%)	6664 (47.63%)	888 (36.39%)	1584 (51.41%)	4192 (49.50%)	
Length of hospital stay, days, median (IQR)	11 (13)	8 (9)	9 (10)	13 (13)	< .001
Comorbidities					
Hypertension, *n* (%)	3881 (27.74%)	404 (16.56%)	925 (30.02%)	2552 (30.13%)	< .001
Diabetes mellitus, *n* (%)	2047 (14.63%)	264 (10.82%)	487 (15.81%)	1296 (15.30%)	< .001
Coronary artery disease, *n* (%)	1378 (9.85%)	89 (3.65%)	321 (10.42%)	968 (11.43%)	< .001
Fracture site					
Upper extremity and trunk fractures^b^, *n* (%)	6185 (44.21%)	2114 (86.64%)	2593 (84.16%)	1478 (17.45%)	< .001
Pelvic fractures, *n* (%)	1074 (7.68%)	0 (0.00%)	0 (0.00%)	1074 (12.68%)	< .001
Lower extremity fractures^c^, *n* (%)	5142 (36.75%)	0 (0.00%)	0 (0.00%)	5142 (60.72%)	< .001
Multiple fractures involving the lower extremities and pelvis^d^, *n* (%)	509 (3.64%)	0 (0.00%)	0 (0.00%)	509 (6.01%)	< .001
Other fractures^e^, *n* (%)	1080 (7.72%)	326 (13.36%)	488 (15.84%)	266 (3.14%)	< .001
Open fracture, *n* (%)	1150 (8.22%)	190 (7.79%)	190 (6.17%)	770 (9.09%)	< .001

Detailed fracture classification criteria are provided in Supplementary Table 1.

SD, standard deviation; IQR, interquartile range.

a *P* values represent overall comparisons among the low-risk and very-low-risk, moderate-risk, and high-risk groups; the overall column was descriptive and was not included in the statistical comparisons.

b Upper extremity and trunk fractures included thoracic and trunk fractures, upper extremity fractures, and combined trunk/upper extremity fractures.

c Lower extremity fractures included femoral fractures, lower leg fractures, patellar fractures, and foot fractures.

d Multiple fractures involving the lower extremities and pelvis included multiple lower extremity fractures, as well as combined pelvic and lower extremity fractures.

e Other fractures included head, facial, and hand fractures.

### 2.4 VTE guidelines and risk stratification

VTE risk assessment was performed using a modified, retrospectively reconstructed Caprini score. In our institution, the Caprini score is routinely used for perioperative VTE risk assessment among surgical patients; therefore, its use in this retrospective cohort reflected local clinical practice. Risk stratification was defined as follows: a score of 0 indicates very low risk; 1-2 indicates low risk (mechanical prophylaxis recommended); 3-4 indicates moderate risk (pharmacological or mechanical prophylaxis recommended); and ≥5 indicates high risk (pharmacological prophylaxis or combined pharmacological and mechanical prophylaxis recommended) [16,17]. Because this retrospective study was based on electronic medical records, the Caprini score was reconstructed from available documented variables. The body mass index (BMI) was excluded from the primary score because height and weight data were missing or unreliable in more than 30% of patients; therefore, the primary score was considered a modified, retrospectively reconstructed Caprini score. To assess the potential impact of excluding BMI, we performed a sensitivity analysis among patients with available BMI and guideline-adherence data by assigning 1 additional Caprini point for BMI > 25 kg/m^2^ and recalculating risk classification, guideline-adherence classification, and area under the curve (AUC). During score reconstruction, surgery-related components were assigned only when the corresponding surgical exposure was documented in the medical record. In patients managed conservatively without surgery, surgery-related items were not assigned. When planned surgery >45 min and elective joint replacement referred to the same surgical procedure, only the higher-scoring elective joint replacement component was assigned, and planned surgery >45 min was not counted separately.

Contraindications to pharmacological prophylaxis were defined according to the Chinese guideline and were categorized as absolute or relative contraindications (Supplementary Table 2). Patients were considered to have a pharmacological prophylaxis contraindication if they had at least one absolute contraindication or two or more relative contraindications. In these patients, mechanical thromboprophylaxis, including graduated compression stockings or intermittent pneumatic compression, was recommended as the initial prophylaxis strategy [16,17].

Patients receiving anticoagulants for possible indications other than thromboprophylaxis could not be reliably distinguished because administration route, dosing frequency, treatment indication, body weight, renal function, and laboratory monitoring were not consistently available; therefore, they remained classified according to the study’s operational prophylaxis definition.

### 2.5 Statistical analysis

Statistical analyses were performed using SPSS software (version 27.0; IBM Corp) and Python 3.12. Descriptive statistics were used to summarize baseline characteristics and to calculate the proportion of patients with recorded thromboprophylaxis use during hospitalization. Continuous variables were summarized as mean ± standard deviation (SD) or median (interquartile range [IQR]), as appropriate, and categorical variables were expressed as frequencies and percentages.

Patients with traumatic fractures were stratified into very low-risk, low-risk, moderate-, and high-risk groups according to the modified Caprini score. The proportion of patients with DVT detected during hospitalization, rates of recorded thromboprophylaxis during hospitalization, and adherence to guideline-recommended prophylaxis were evaluated across these groups. For the operational guideline-adherence analysis, adherence was assessed only among patients for whom routine thromboprophylaxis was recommended; therefore, patients in the very-low-risk group (modified Caprini score 0) were excluded. In addition, among patients with DVT, those whose recorded pharmacological or mechanical prophylaxis was initiated only after DVT detection were excluded from the adherence denominator because these measures could not be considered pre-detection prophylaxis. Based on the recorded pharmacological and mechanical prophylaxis indicators, prophylaxis practice was categorized as no recorded prophylaxis, mechanical prophylaxis only, pharmacological prophylaxis only, or combined pharmacological and mechanical prophylaxis; timing was used only to determine whether prophylaxis was initiated before or after DVT detection. In patients with contraindications to pharmacological prophylaxis, mechanical prophylaxis alone or mechanical prophylaxis followed by pharmacological prophylaxis was considered guideline-concordant, whereas pharmacological prophylaxis alone or pharmacological prophylaxis started before or simultaneously with mechanical prophylaxis was considered non-concordant. In addition, univariate and multivariable logistic regression analyses were performed for variables included in the Caprini model.

In addition, the types of anticoagulant agents used were analyzed. Variables included in the reconstructed modified Caprini score were first assessed using univariate logistic regression. Variables with *P* < .05 in univariate analysis were considered for entry into the multivariable logistic regression model. Variables with fewer than 10 DVT events among exposed patients were not included in the final multivariable model to reduce sparse-data bias and unstable odds-ratio estimates. Predictors were coded according to the corresponding modified Caprini components, generally as binary variables indicating the presence or absence of each risk factor; age was categorized according to the modified Caprini age groups. Multicollinearity was assessed using variance inflation factors, and strongly collinear variables were not entered simultaneously into the final model. Length of hospital stay was not included in the multivariable model because it may represent both a consequence of DVT detection and the duration of the observation window rather than a baseline predictor. The multivariable logistic regression analysis was used to identify factors associated with in-hospital detected DVT rather than to develop a new clinical prediction model. Therefore, model-derived AUC comparisons were not performed. The discriminative ability of the modified Caprini score for in-hospital detected DVT was assessed descriptively using receiver operating characteristic (ROC) curve analysis, and the AUC was calculated. The optimal cutoff was explored using the Youden index; this analysis was exploratory and was not intended to redefine guideline-based risk categories. Because no new prediction model was developed, formal model derivation, internal validation, and calibration assessment were not performed. A 2-sided *P*-value < .05 was considered statistically significant.

## 3 Results

### 3.1 Patient demographic and clinical characteristics

A total of 14,887 patient records were initially identified during the predefined study period. Of these, 224 were excluded because the patients were younger than 18 years, and 673 were excluded because diagnostic information was insufficient to confirm traumatic fracture. Consequently, 13,990 patients were included in the final analysis, comprising 7326 men and 6664 women. The mean age was 61.0 ± 17.9 years.

Thoracic and trunk fractures and femoral fractures accounted for the largest proportions of cases (26.23% and 21.12%, respectively). In addition, 1150 patients presented with open fractures. The prevalence of hypertension, diabetes mellitus, and coronary artery disease was significantly higher in the moderate-risk and high-risk groups compared with the low-risk group (all *P* < .001). Furthermore, the length of hospital stay increased progressively with higher risk categories (Table 1).

### 3.2 In-hospital DVT detection

A total of 13,990 eligible patients with traumatic fractures were included in the final analysis. Among them, DVT was detected during hospitalization in 1765 patients (12.62%). Proximal involvement was documented in 456 patients (25.84%), whereas 1309 patients (74.16%) had isolated distal DVT. In addition, 162 patients developed pulmonary embolism (1.16%). Stratified analyses showed that the proportion of patients with in-hospital detected DVT was significantly higher in patients with lower-extremity and pelvic fractures than in those with fractures at other sites. The highest proportion was observed in patients with multiple fractures involving the lower extremities and pelvis (31.83%) (Table 2). Among patients with in-hospital detected DVT, 150 patients (8.50%) had no recorded pharmacological or mechanical prophylaxis, 87 (4.93%) received mechanical prophylaxis only, 709 (40.17%) received pharmacological prophylaxis only, and 819 (46.40%) received combined pharmacological and mechanical prophylaxis. At the patient level, at least one prophylactic measure had been initiated before DVT detection in 1093 patients (61.93%), whereas all recorded prophylactic measures were initiated only after DVT detection in 522 patients (29.58%). These findings indicate that recorded thromboprophylaxis use did not consistently represent pre-detection exposure; therefore, the 522 patients whose prophylaxis was initiated only after DVT detection were excluded from the operational guideline-adherence analysis (Supplementary Table 3).

**Table 2:** DVT detection according to fracture characteristics and recorded thromboprophylaxis practice during hospitalization

Characteristic	In-hospital DVT detection^a^, *n/N* (%)	*P* value
Overall (*N* = 13,990)	1765/13,990 (12.62%)	
Fracture site		< .001
Thoracic and trunk fractures (*n* = 3669)	291/3669 (7.93%)	
Upper extremity fractures (*n* = 2107)	72/2107 (3.42%)	
Combined thoracic/trunk and upper extremity fractures (*n* = 409)	39/409 (9.54%)	
Pelvic fractures (*n* = 1074)	169/1074 (15.74%)	
Femoral fractures (*n* = 2955)	654/2955 (22.13%)	
Lower leg fractures (*n* = 1432)	202/1432 (14.11%)	
Patellar fractures (*n* = 534)	64/534 (11.99%)	
Foot fractures (*n* = 221)	28/221 (12.67%)	
Multiple fractures involving the lower extremities and pelvis (*n* = 509)	162/509 (31.83%)	
Other fractures^b^ (*n* = 1080)	84/1080 (7.78%)	
Fracture type		< .001
Open fractures (*n* = 1150)	184/1150 (16.00%)	
Closed fractures (*n* = 12,840)	1581/12,840 (12.31%)	
Recorded thromboprophylaxis practice		< .001
No recorded prophylaxis	150/5264 (2.85%)	
Mechanical prophylaxis only	87/2714 (3.21%)	
Pharmacological prophylaxis only	709/1976 (35.88%)	
Combined pharmacological and mechanical prophylaxis	819/4036 (20.29%)	

Recorded thromboprophylaxis practice refers to prophylaxis documented at any time during hospitalization and does not necessarily indicate prophylaxis exposure before deep vein thrombosis detection. Therefore, comparisons involving prophylaxis practice are descriptive and should not be interpreted causally. Detailed timing of prophylaxis initiation relative to deep vein thrombosis detection is shown in Supplementary Table 3.

DVT, deep vein thrombosis.

a In-hospital DVT was defined as ultrasound-confirmed in-hospital lower-extremity DVT, including both proximal and distal DVT. Pulmonary embolism was reported separately and was not included in the DVT outcome.

b Other fractures included head, facial, and hand fractures.

### 3.3 Assessment of thrombosis risk in hospitalized patients with traumatic fractures

#### 3.3.1 Modified caprini score

According to the modified Caprini score, 8469 patients (60.54%) were classified as high risk, 3081 (22.02%) as moderate risk, and 2440 (17.44%) as low or very low risk.

Univariate logistic regression was performed for variables included in the modified Caprini score, and variables with *P* < .05 were considered for multivariable logistic regression (Supplementary Table 4). Variables with fewer than 10 DVT events among exposed patients, including acute myocardial infarction and elevated serum homocysteine, were not entered into the final multivariable model because of sparse data. Because hip, pelvic, or lower limb fracture was highly correlated with reduced mobility >72 hours (r = 0.972), these 2 variables were not entered simultaneously; reduced mobility >72 hours was retained in the final model. After these exclusions, no substantial multicollinearity was observed, with a maximum VIF of 1.35. In the final multivariable model, age >75 years, severe pulmonary disease or abnormal pulmonary function, lower extremity varicose veins, reduced mobility >72 hours, planned surgery >45 min, central venous catheterization, history of VTE, and acute spinal cord injury remained independently associated with in-hospital detected DVT (Table 3). Among these, history of VTE was the strongest predictor, with an adjusted OR of 5.56 (95% CI, 3.44-8.98; *P* < .001).

**Table 3:** Multivariable logistic regression of modified Caprini components associated with in-hospital detected DVT

Variable	OR (95% CI)	*P* value
Age 41–60 y	1.07 (0.94-1.22)	.284
Age >75 y	1.91 (1.67-2.17)	< .001
Lower extremity edema	1.18 (0.96-1.46)	.115
Severe pulmonary disease/abnormal pulmonary function	1.61 (1.42-1.82)	< .001
Sepsis	1.58 (0.98-2.55)	.060
Lower extremity varicose veins	2.92 (1.34-6.37)	.007
Reduced mobility >72 h	3.05 (2.70-3.45)	< .001
Malignant neoplasm	0.83 (0.66-1.04)	.108
Planned surgery >45 min	1.86 (1.65-2.09)	< .001
Plaster cast	0.91 (0.73-1.14)	.413
Central venous catheter	1.91 (1.51-2.40)	< .001
History of VTE	5.56 (3.44-8.98)	< .001
Elective joint replacement	0.75 (0.62-0.90)	.003
Stroke within 1 month	1.10 (0.91-1.31)	.322
Acute spinal cord injury	1.68 (1.27-2.21)	< .001

Variables with fewer than 10 DVT events among exposed patients, including acute myocardial infarction and elevated serum homocysteine, were not entered into the final multivariable model. Hip, pelvic, or lower limb fracture was excluded because of strong collinearity with reduced mobility >72 hours.

CI, confidence interval; DVT, deep vein thrombosis; OR, odds ratio; VTE, venous thromboembolism.

**Figure 1:**
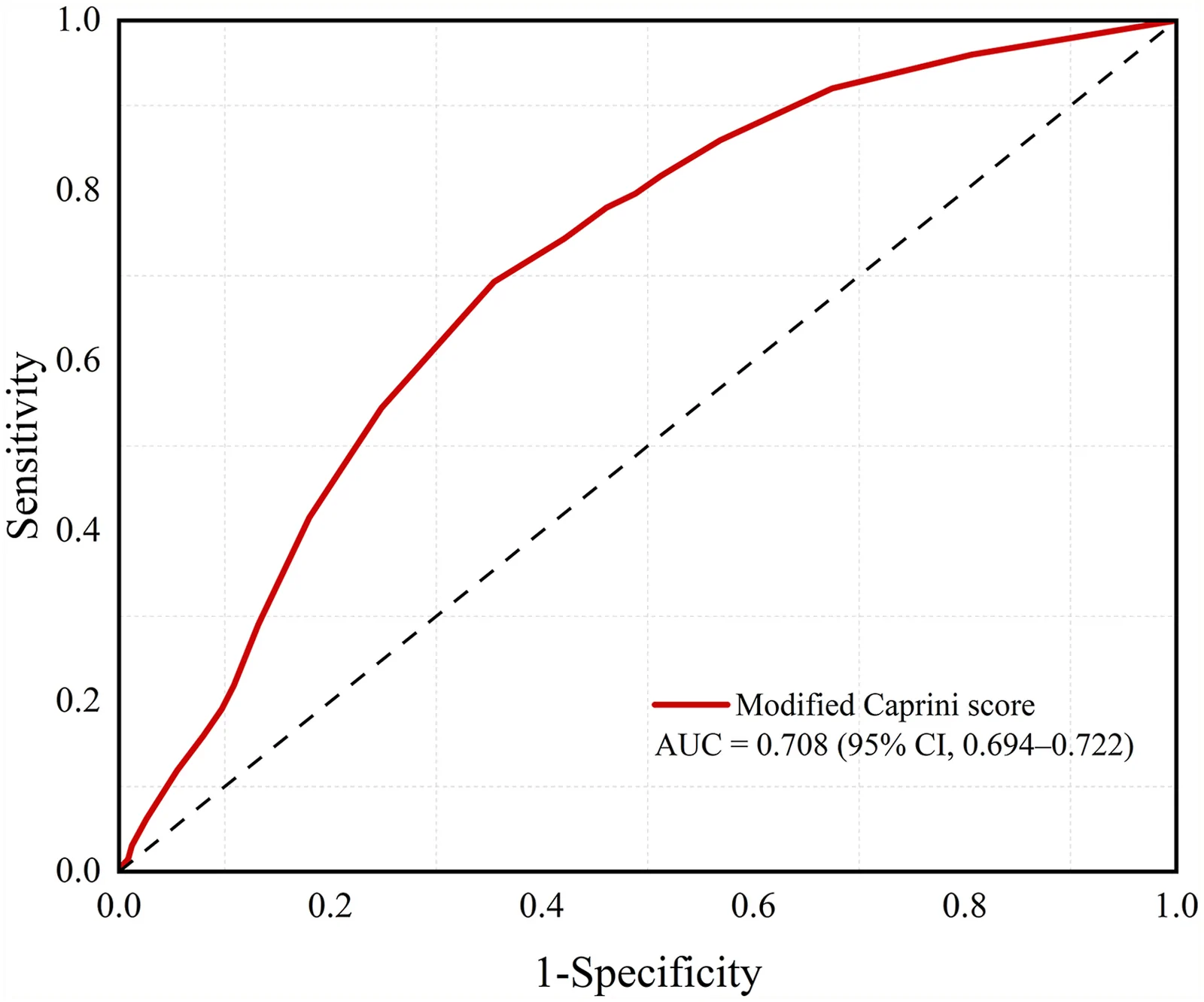
Receiver operating characteristic curve of the modified Caprini score for in-hospital detected DVT. The ROC curve was generated using the modified Caprini total score in the overall cohort. The AUC was 0.708 (95% CI, 0.694-0.722). The Youden-derived optimal cutoff was ≥10, with a sensitivity of 69.3% and specificity of 64.5%. DVT, deep vein thrombosis; ROC, receiver operating characteristic; AUC, area under the curve.

ROC curve analysis was performed to evaluate the discriminative performance of the modified Caprini score for in-hospital detected DVT. The AUC was 0.708 (95% CI, 0.694-0.722), indicating moderate discrimination (Figure 1). The Youden-derived optimal cutoff was a modified Caprini score of ≥10, with a sensitivity of 69.3% and specificity of 64.5%. At the conventional high-risk cutoff of ≥5, sensitivity was higher (85.9%) but specificity was lower (43.1%). This cutoff analysis was exploratory and was not intended to redefine guideline-based risk categories.

In the BMI sensitivity analysis, 5814 patients had both BMI and guideline-adherence data. After adding 1 point for BMI >25 kg/m^2^, 335 patients (5.76%) changed risk category and 60 patients (1.03%) changed guideline-adherence classification; the overall guideline-concordant rate changed from 53.03% to 52.27%, and the AUC changed from 0.702 to 0.697 (Supplementary Table 5).

#### 3.3.2 High-risk group

Among the 8469 patients classified as high risk, DVT was detected during hospitalization in 1517 patients (17.91%). A total of 6465 patients (76.34%) had recorded thromboprophylaxis use during hospitalization, including 1418 patients (16.74%) who received mechanical prophylaxis only, 1601 patients (18.90%) who received pharmacological prophylaxis only, and 3446 patients (40.69%) who received combined pharmacological and mechanical prophylaxis. Overall, 5047 patients (59.59%) received pharmacological thromboprophylaxis, either alone or in combination with mechanical prophylaxis. Among high-risk patients with in-hospital detected DVT, mechanical prophylaxis was recorded in 815 patients and pharmacological prophylaxis in 1349 patients; initiation occurred after DVT detection in 474 and 478 patients, respectively (Supplementary Table 3).

In addition, 1100 patients (12.99%) had at least one absolute contraindication or 2 or more relative contraindications to pharmacological prophylaxis. Notably, 299 of these patients (27.18%) still received pharmacological prophylaxis within 24 hours of admission (Supplementary Table 6).

#### 3.3.3 Moderate-Risk Group

Among the 3081 patients classified as moderate risk, DVT was detected during hospitalization in 177 patients (5.74%). A total of 1522 patients (49.40%) had recorded thromboprophylaxis use during hospitalization, including 814 patients (26.42%) who received mechanical prophylaxis only, 261 patients (8.47%) who received pharmacological prophylaxis only, and 447 patients (14.51%) who received combined pharmacological and mechanical prophylaxis. Overall, 708 patients (22.98%) received pharmacological prophylaxis, either alone or in combination with mechanical prophylaxis. Among moderate-risk patients with in-hospital detected DVT, mechanical prophylaxis was recorded in 70 patients and pharmacological prophylaxis in 130 patients; initiation occurred after DVT detection in 30 and 50 patients, respectively (Supplementary Table 3).

Furthermore, 404 patients (13.11%) had at least one absolute contraindication or two or more relative contraindications to pharmacological prophylaxis. Notably, 81 of these patients (20.05%) still received pharmacological prophylaxis within 24 hours of admission (Supplementary Table 7). These findings reflect observed real-world prescribing practice; pharmacological prophylaxis given in the presence of pharmacological contraindications was not considered guideline-concordant unless it followed mechanical prophylaxis.

#### 3.3.4 Low-risk and very low-risk groups

Among the 2440 patients classified as low or very low risk, DVT was detected during hospitalization in 71 patients (2.91%). Specifically, 62 cases (2.91%) occurred in the low-risk group and 9 cases (2.88%) in the very low-risk group. During hospitalization, 1701 patients (69.71%) received no recorded prophylaxis, 482 patients (19.75%) received mechanical prophylaxis only, 114 patients (4.67%) received pharmacological prophylaxis only, and 143 patients (5.86%) received combined pharmacological and mechanical prophylaxis (Supplementary Table 8). Among low-risk and very low-risk patients with in-hospital detected DVT, mechanical prophylaxis was recorded in 21 patients and pharmacological prophylaxis in 49 patients; initiation occurred after DVT detection in 3 and 15 patients, respectively (Supplementary Table 3).

### 3.4 Anticoagulant use

Among patients receiving pharmacological thromboprophylaxis, low-molecular-weight heparins, including enoxaparin, nadroparin, and dalteparin, were the most commonly used agents (87.48%), followed by unfractionated heparin (7.15%). Oral anticoagulants, such as rivaroxaban and edoxaban, were used less frequently (5.37%). Among the low-molecular-weight heparins, enoxaparin was the most frequently prescribed agent (65.89%), followed by nadroparin (17.74%) and dalteparin (16.37%) (Supplementary Table 9).

### 3.5 Prevention rate and guideline adherence

**Figure 2:**
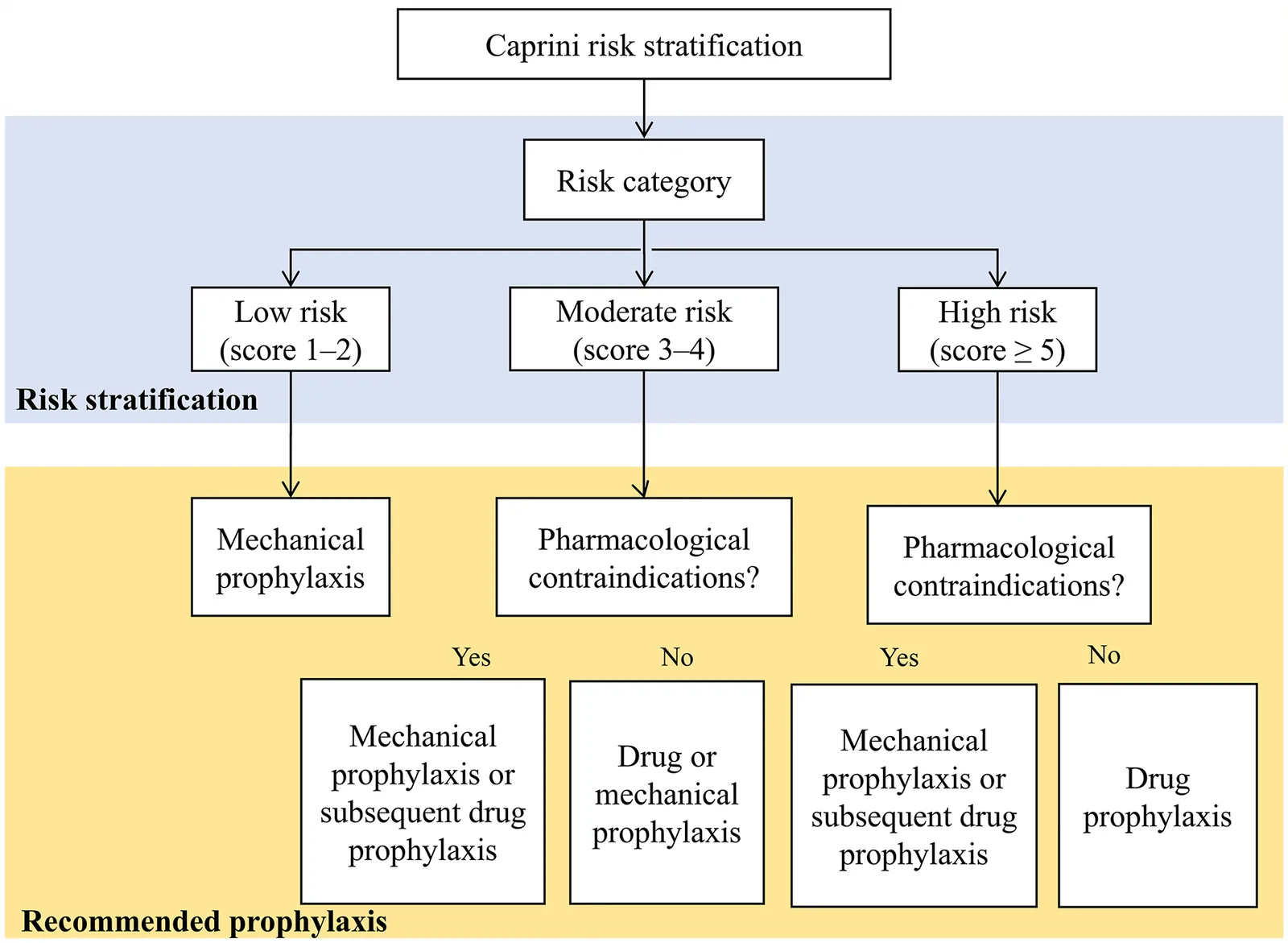
Guideline-based thromboprophylaxis recommendations according to Caprini risk stratification. Patients were stratified as low risk (score 1-2), moderate risk (score 3-4), and high risk (score ≥5). Drug prophylaxis refers to pharmacological prophylaxis. Mechanical prophylaxis was recommended for low-risk patients. For moderate-risk patients without contraindications to pharmacological prophylaxis, drug or mechanical prophylaxis was considered guideline-concordant. For high-risk patients without contraindications, drug prophylaxis was recommended. For patients with contraindications to pharmacological prophylaxis, mechanical prophylaxis alone or mechanical prophylaxis followed by subsequent drug prophylaxis was considered guideline-concordant.

Based on the guideline recommendations illustrated in Figure 2, the rates of thromboprophylaxis and guideline adherence were evaluated in hospitalized patients with traumatic fractures. The operational guideline-adherence analysis was restricted to patients for whom routine thromboprophylaxis was recommended. After excluding patients in the very-low-risk group and patients whose recorded prophylaxis was initiated only after DVT detection, 13,156 patients were included in the operational guideline-adherence analysis. Among these patients, 8121 (61.73%) received recorded pharmacological or mechanical thromboprophylaxis, and 6065 (46.10%) received guideline-concordant prophylaxis. The detailed operational classification is presented in Supplementary Table 10.

**Figure 3:**
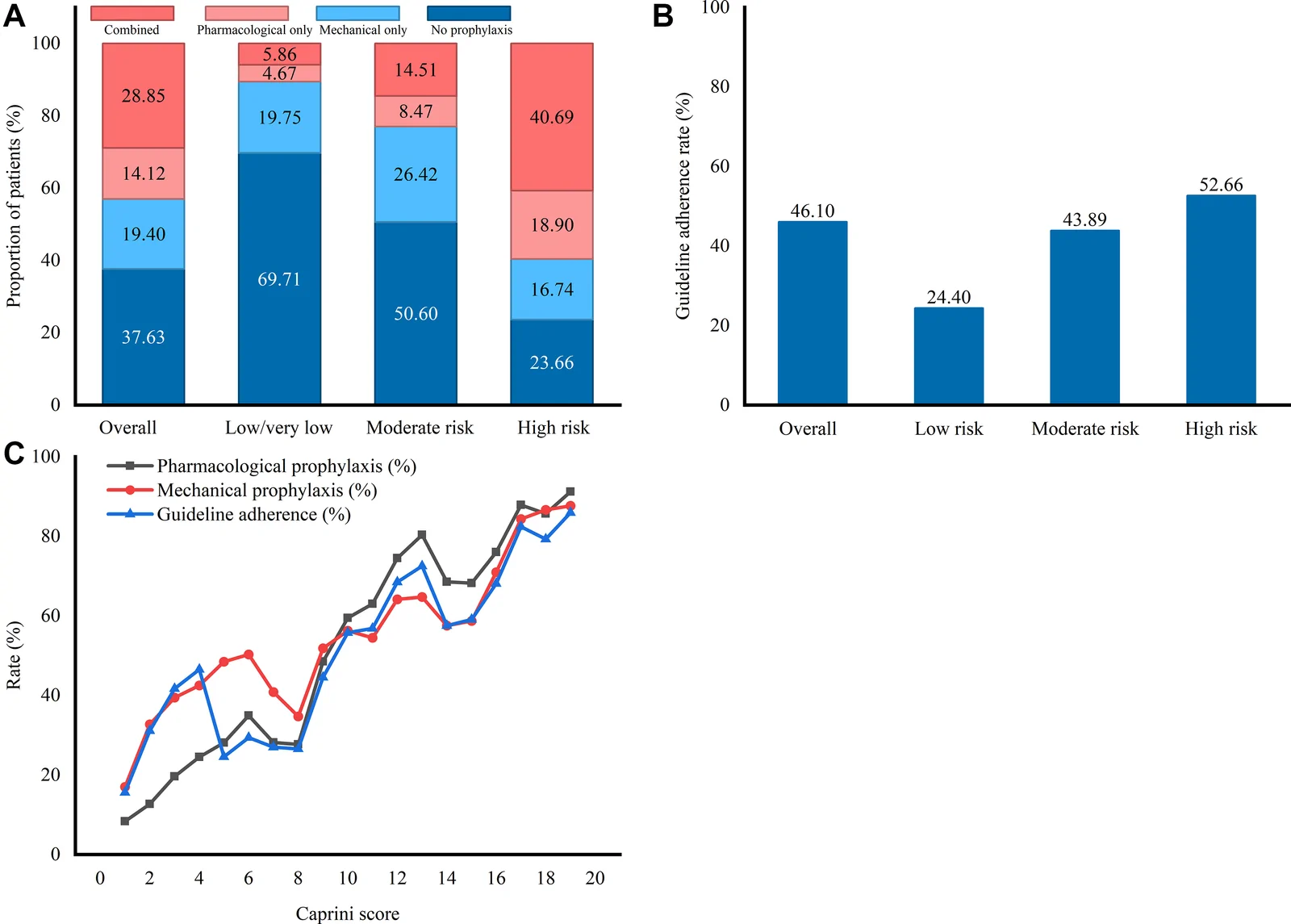
Thromboprophylaxis patterns and guideline adherence according to risk category and modified Caprini score. (A) Distribution of recorded thromboprophylaxis patterns during hospitalization in the overall cohort and across modified Caprini risk categories. Recorded prophylaxis was categorized as no prophylaxis, mechanical prophylaxis only, pharmacological prophylaxis only, or combined pharmacological and mechanical prophylaxis. (B) Guideline-adherence rates in the operational guideline-adherence analysis population. For the operational guideline-adherence analysis, adherence was assessed only among patients for whom routine thromboprophylaxis was recommended; therefore, patients in the very-low-risk group (modified Caprini score 0) were excluded. Patients whose pharmacological or mechanical prophylaxis was initiated only after DVT detection were also excluded because these measures could not be considered pre-detection prophylaxis. Therefore, the denominators in panel B differ from those in panel A. (C) Rates of pharmacological prophylaxis, mechanical prophylaxis, and guideline adherence according to modified Caprini score in the operational guideline-adherence analysis population. The analysis was restricted to patients for whom routine thromboprophylaxis was recommended. Patients with a modified Caprini score ≥20 were grouped together.

Stratified analyses showed that 516 low-risk patients (24.40%) received guideline-recommended prophylaxis. Among moderate-risk patients, 1328 (43.89%) received appropriate pharmacological or mechanical prophylaxis, while 4221 high-risk patients (52.66%) received appropriate thromboprophylaxis. The proportion of patients receiving appropriate thromboprophylaxis increased across risk categories and was significantly higher in the moderate- and high-risk groups than in the low-risk group (*P* < .001) (Table 4; Figure 3A, B). Furthermore, the rates of mechanical prophylaxis, pharmacological prophylaxis, and guideline adherence generally increased with increasing modified Caprini scores (Figure 3C).

**Table 4:** Adherence to thromboprophylaxis guidelines in hospitalized patients with traumatic fractures

Risk group	Number of patients	Guideline adherence, *n/N* (%)	*P* value
Overall^a^	13,156	6065/13,156 (46.10%)	< .001
Low-risk group	2115	516/2115 (24.40%)	
Moderate-risk group	3026	1328/3026 (43.89%)	
High-risk group	8015	4221/8015 (52.66%)	

DVT, deep vein thrombosis.

a Overall indicates the population included in the operational guideline-adherence analysis. For the operational guideline-adherence analysis, adherence was assessed only among patients for whom routine thromboprophylaxis was recommended; therefore, patients in the very-low-risk group (modified Caprini score 0) were excluded. Patients whose pharmacological or mechanical prophylaxis was initiated only after DVT detection were also excluded because these measures could not be considered pre-detection prophylaxis.

## 4 Discussion

In this study, DVT was detected during hospitalization in 12.62% of patients with traumatic fractures, whereas only 46.10% of patients in the operational adherence analysis received guideline-recommended thromboprophylaxis. These results indicate a substantial burden of in-hospital detected DVT and suboptimal adherence to thromboprophylaxis guidelines in this population, consistent with previous reports [9,18,19]. Given the potential complications of DVT, including pulmonary embolism and post-thrombotic syndrome, appropriate thromboprophylaxis remains particularly important in high-risk patients [20]. However, the coexistence of high in-hospital DVT detection and suboptimal guideline adherence should not be interpreted as evidence that nonadherence caused the observed DVT burden.

Furthermore, the modified Caprini score demonstrated moderate discriminative performance for in-hospital detected DVT, with an AUC of 0.708 (95% CI, 0.694-0.722). This finding suggests that the modified Caprini score retained discriminative value in this traumatic fracture population, rather than indicating poor performance. Previous studies in orthopedic and trauma-related populations have also supported the usefulness of Caprini-based risk assessment, although reported performance may vary according to patient population, outcome definition, timing of DVT assessment, and ultrasound screening strategy [9,21]. Although the exploratory Youden-derived cutoff was ≥10, 60.54% of patients were classified as high risk using the conventional ≥5 cutoff, which showed high sensitivity but relatively low specificity. Therefore, the application of this conventional threshold in traumatic fracture patients should be interpreted cautiously.

Current guidelines, such as those from the American College of Chest Physicians, do not specifically address patients with traumatic fractures, and recommendations are often extrapolated from general surgical populations [22]. However, patients with traumatic fractures appear to have a higher risk of VTE than general surgical patients, indicating that more tailored risk assessment and prevention strategies may be beneficial in this population [4,23].

In this context, our findings suggest that Caprini-based risk assessment may require further refinement for patients with traumatic fractures, consistent with previous studies [8,9]. Trauma-specific pathophysiological features, including acute inflammation, tissue injury, prolonged immobilization, and trauma-related coagulation abnormalities, may not be fully captured by the current scoring system. In addition, because the Caprini score was retrospectively reconstructed from electronic medical records and BMI was excluded due to missing or unreliable height and weight data, scores may have been underestimated in some patients. These limitations may have affected risk classification, guideline-adherence assessment, and predictive performance; therefore, the Caprini-related findings should be interpreted in the context of this modified score.

Multivariable analysis identified several modified Caprini components independently associated with in-hospital detected DVT, including history of VTE, reduced mobility >72 hours, central venous catheterization, planned surgery >45 min, age >75 years, severe pulmonary disease or abnormal pulmonary function, lower extremity varicose veins, and acute spinal cord injury. Among these, history of VTE showed the strongest association, consistent with previous studies [24].

These findings emphasize the importance of thorough clinical history-taking and risk assessment. However, incomplete documentation of prior VTE in medical records may lead to underestimation of risk and inadequate thromboprophylaxis in clinical practice.

Regarding guideline adherence, fewer than half of patients in the operational adherence analysis received guideline-concordant prophylaxis after excluding very-low-risk patients and patients whose prophylaxis was initiated only after DVT detection. This rate was lower than that reported in some previous studies, suggesting persistent gaps between guideline recommendations and real-world clinical practice [25].

Several factors may contribute to this discrepancy, including limited awareness of guidelines, underestimation of VTE risk, and concerns regarding bleeding complications. The higher observed proportion of DVT among patients recorded as receiving pharmacological thromboprophylaxis should be interpreted cautiously, as it may reflect confounding by indication and initiation or escalation of anticoagulation after DVT detection. Therefore, these crude comparisons were considered descriptive and were not used to infer the protective or harmful effect of prophylaxis. Accordingly, suboptimal guideline adherence should not be interpreted as a causal explanation for the high burden of in-hospital detected DVT.

### 4.1 Limitations

This study has several limitations. First, as a retrospective study based on electronic medical records, data quality depended on the accuracy and completeness of the original documentation, and missing data were more common than in prospectively collected datasets. The Caprini score was retrospectively reconstructed using available documented variables, and BMI could not be included because height and weight data were incomplete or unreliable in a substantial proportion of patients. Because BMI is a component of the original Caprini model, its exclusion may have underestimated risk in some patients. However, in the sensitivity analysis restricted to patients with available BMI, adding the BMI component changed risk classification in only 5.76% of patients and guideline-adherence classification in 1.03%, suggesting that the primary conclusions were not materially altered. Nevertheless, because BMI was unavailable for a substantial proportion of patients, residual bias related to missing BMI data cannot be excluded. In addition, severe traumatic brain injury was identified primarily according to diagnostic coding; however, in clinical practice, its assessment also depends on the patient’s level of consciousness and Glasgow Coma Scale score. Therefore, misclassification may still have been present.

Second, the temporal relationships among DVT onset, DVT detection, and thromboprophylaxis initiation could not be fully established. DVT detection time was defined as the time of the first ultrasonography examination showing DVT, whereas prophylaxis initiation time was defined using the first recorded medical order. However, detection time did not necessarily correspond to thrombus onset, and some patients initiated pharmacological or mechanical prophylaxis only after DVT had already been detected. To reduce reverse causation in the guideline-adherence analysis, patients whose prophylaxis was initiated only after DVT detection were excluded from the adherence denominator. Moreover, because ultrasonography was not performed systematically at admission and thrombus chronicity could not be reliably established, some DVTs detected during hospitalization may have predated admission. Therefore, the present study could neither determine the timing of DVT onset nor estimate the causal effect of thromboprophylaxis on DVT occurrence.

Third, the single-center retrospective design may limit generalizability. Institutional ultrasonography and thromboprophylaxis practices, together with variability in documentation quality, may have influenced both DVT ascertainment and apparent guideline adherence. Lower-extremity venous ultrasonography was not performed universally at admission or systematically throughout hospitalization; rather, it was generally prompted by symptoms or clinical suspicion. Consequently, asymptomatic DVT may have gone undetected, whereas patients perceived to be at higher risk may have been more likely to undergo imaging. If the likelihood of ultrasonographic assessment differed across modified Caprini risk categories or groups defined by recorded prophylaxis practice, comparisons of DVT detection proportions across these groups may have been biased by differential outcome ascertainment. Thus, the observed 12.62% represents the proportion of patients with DVT detected under routine clinical practice and should not be interpreted as an incidence estimate derived from systematic screening. Residual confounding cannot be excluded. In addition, we did not perform formal internal validation or calibration analyses; therefore, the ROC results should be interpreted as a descriptive assessment of discrimination rather than as validation of a new prediction model.

Finally, therapeutic anticoagulation for indications other than thromboprophylaxis, such as atrial fibrillation or previous VTE, could not be reliably distinguished from prophylactic anticoagulation in some patients because medication-order data did not consistently include the route or frequency of administration, body weight, renal function, laboratory monitoring, or a documented indication for anticoagulation. Warfarin was not included as pharmacological thromboprophylaxis in the operational definition. Consequently, some patients receiving treatment-intensity anticoagulation or extended secondary prevention may have been classified as receiving pharmacological thromboprophylaxis, potentially leading to exposure misclassification and residual confounding in analyses of DVT detection and guideline adherence.

## 5 Conclusion

Approximately one in 8 patients with traumatic fractures had DVT detected during hospitalization. The modified Caprini score showed moderate discriminative ability for in-hospital detected DVT, although its clinical application in patients with traumatic fractures should be interpreted cautiously because a large proportion of patients were classified as high risk.

In addition, only 46.10% of patients in the operational guideline-adherence analysis received guideline-recommended thromboprophylaxis, indicating a substantial gap between guideline recommendations and real-world clinical practice. These findings indicate that a high burden of in-hospital detected DVT coexisted with suboptimal guideline adherence in this single-center real-world cohort. Because thromboprophylaxis could be initiated either before or after DVT detection, the present findings should be interpreted as describing real-world prophylaxis practice rather than estimating the causal effect of prophylaxis on DVT risk. Addressing these issues may help improve real-world VTE prevention practice in patients with traumatic fractures.
